# Metastasis of Ewing Sarcoma to the Pancreas: Case Report and Literature Review

**DOI:** 10.1155/2020/7075048

**Published:** 2020-03-21

**Authors:** Hyma Polimera, Prashanth Moku, Shady Piedra Abusharar, Monali Vasekar, Jayakrishna Chintanaboina

**Affiliations:** Penn State Hershey Medical Center, Hershey, Pennsylvania 17033, USA

## Abstract

Ewing sarcoma (ES) is a highly aggressive malignant bone cancer. ES is part of the Ewing sarcoma family of tumors (ESFT), which express characteristic t(11;22) translocation as well as higher levels of CD99. Given that metastasis and tumor burden are significant prognostic factors in patient's response to treatment, prompt diagnosis is needed to effectively treat ESFT patients. However, the challenges in classifying and characterizing ESFT complicate effective management and treatment of ES. In this report, we present a rare case of ES metastasis to the pancreas. Upon review of the literature, we found 39 cases of ESFT involving the pancreas, but only 3 were metastatic to the pancreas while the remaining cases of ESFT primarily originated from the pancreas. Given the rarity of such metastasis, the positive outcome in our patient's case may explain the importance of prompt diagnosis in order to initiate appropriate treatment.

## 1. Introduction

Ewing sarcoma (ES) is highly aggressive and is the most common malignant bone cancer in children and young adults after osteosarcoma [1]. ES was first described as an osteolytic bone tumor composed of malignant, small round cells, by James Ewing in 1921, and extraosseous ES was first described by Tefft in 1969.

A major marker for Ewing sarcoma is the presence of t(11;22) chromosomal translocation [1]. Primitive neuroectodermal tumors (PNET) also have a similar t(11;22) chromosomal translocation [1]. The translocation yields an EWS-FLI1 fusion protein, which acts as an oncogenic transcription factor. ES and PNET are part of the Ewing sarcoma family of tumors (ESFT). Specifically, the Ewing sarcoma family of tumors are comprised of Ewing sarcoma of the bone (ESB), extraosseous Ewing sarcoma (EES), peripheral primitive neuroectodermal tumor (pPNET), and Askin tumor (a pPNET that originates in the chest wall) [2]. In addition to the characteristic t(11;22) translocation, the Ewing sarcoma family of tumors also expresses significantly higher levels of CD99 (MIC2) protein than observed in normal tissue [3]. Overall, the Ewing sarcoma family of tumors exhibit similar morphological, immunophenotypic, cytogenetic, and histological features.

The ESFT also shares certain clinical characteristics, such as a peak incidence during the teenage years, a tendency to spread rapidly, and responsiveness to the same chemotherapeutic regimens and radiation therapy. Effective management of ESFT requires prompt diagnosis, but characterization and classification is very challenging. Nearly 30% of patients have clinical metastatic disease at the time of diagnosis, and these patients have particularly poor prognosis despite aggressive treatment regimen [4].

Furthermore, metastasis and tumor burden are significant prognostic factors in determining a patient's response to treatment. While Ewing sarcoma is a primary malignancy of the bone, EES/pPNETs often originate in the soft tissue of thoraco-pulmonary, pelvic, and lower extremity regions, but rarely from the pancreas [5].

In this case report, we present a 29-year-old man with cytogenetically confirmed metastatic EES to the pancreas.

## 2. Case Presentation

A 29-year-old male, with a past medical history of extraosseous Ewing sarcoma of the right triceps, presented for a follow-up of his disease and was found to have an enlarged gastrohepatic lymph node.

He was initially diagnosed with ES of the right triceps (T2b N0 M0) at 12 years of age. He was treated with standard chemotherapy with VAC (vincristine, adriamycin, and cyclophosphamide) alternating with IE (ifosfamide and etoposide). He was then treated with radiation therapy consisted of 4500 cGy (180 cGy/fraction) with a boost to residual gross disease of 200 cGy/fraction for a total dose of 5500 cGy. The residual mass was followed thereafter, and he remained in remission until he turned 26 years.

At the age of 26 years, the patient had local recurrence and was found to have an increase in size of the mass within the right proximal triceps and abutting the humerus. FNA and core biopsy was positive for a small blue cell tumor and positive for CD99. These findings were consistent with Ewing sarcoma. He was then treated with temozolomide/irinotecan × 2; cyclophosphamide and topotecan × 6 followed by limb-sparing resection of his right upper arm mass with negative margins (closest 2 mm), with no further evidence of local recurrence.

**Figure 1:**
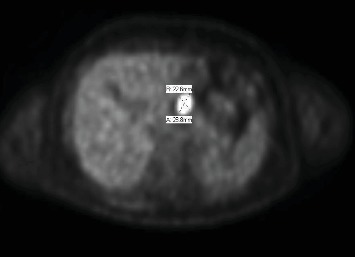
PET scan: gastrohepatic lymph node that measures 2.8 × 2.3 cm.

Nearly 2.5 years after the aforementioned episode of local recurrence, the patient developed metastatic recurrence in his lungs and right orbit. Radiographic studies showed 3 nodules in the right lung and a mass in the right greater sphenoid that extended into his right orbit. An enlarged hepatic lymph node was also noted (Figure 1). CT-guided biopsy of the lung nodule was positive for malignant cells consistent with metastatic Ewing sarcoma. The patient subsequently received radiation therapy (30 Gy/10 fx) to his right orbit for his sphenoid lesion and 8 courses of cyclophosphamide and topotecan.

**Figure 2:**
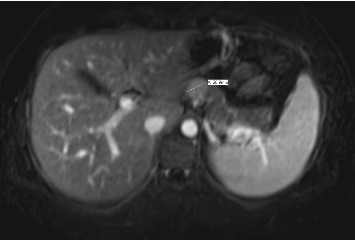
MRI of the abdomen: 2.1 cm slightly T2 hyperintense, T1 hypointense nodule in the pancreatic neck.

**Figure 3:**
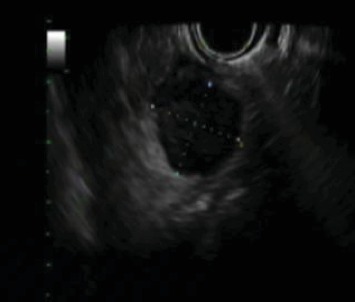
Upper EUS: 18 × 16 mm lesion in the pancreatic body.

Follow-up imaging after completion of the therapy showed good response of lung metastasis with complete resolution of 2 nodules previously presented and near complete response of the 3^rd^ nodule. Imaging also demonstrated orbital disease responded well to therapy. However, the patient's abdominal mass had minimal response. Positron emission tomography (PET) scan remained positive for the aforementioned mass, thought to be a gastrohepatic lymph node. An MRI of the abdomen subsequently showed a 2.1 cm mass in the pancreatic neck (Figure 2). An endoscopic ultrasound (EUS) with fine-needle aspiration was performed for further evaluation. EUS showed an 18 × 16 mm hypoechoic mass in the proximal body of the pancreas (Figure 3). Cytology of the fine-needle aspirate of the mass showed malignant cells consistent with Ewing sarcoma. Furthermore, tissue sections demonstrated a small blue cell tumor. The tumor cells were positive for CD99 but negative for both CD45 and S100, which was consistent with Ewing sarcoma.

**Figure 4:**
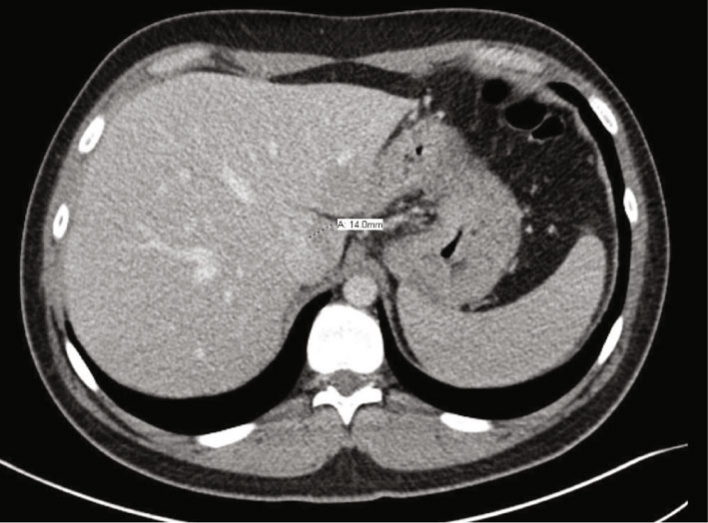
CT of the abdomen: significant regression of the metastatic nodular lesion to 1.4 cm in the pancreatic neck following radiation therapy.

After discussion at the Sarcoma Tumor Board Conference at our institution and based on his previous successful response to radiation therapy to his right orbit, he was treated with radiation (30 Gy/10 fx) to the pancreatic metastasis. There was significant regression of the pancreatic mass after radiation therapy based on the follow-up CT imaging (Figure 4). He remained symptom-free and disease-free at follow-up after 12 months and is currently undergoing periodic surveillance. The follow-up protocol typically followed includes MRI/CT with contrast of primary site, and chest imaging (CT or X-ray) every 3 months for 2 years, every 6-12 from 2 to 5 years, and annually thereafter.

## 3. Discussion

Ewing sarcoma is a rare tumor that involves bones or soft tissue surrounding the bone. Five-year overall survival (OS) in patients with ESFT varies significantly based on the presence of metastasis at diagnosis. Patients with metastatic disease have a reported five-year overall survival between 9 and 41% whereas patients with localized disease have a five-year OS approximately 70% [6]. A recent study on 281 patients with primary disseminated multifocal Ewing sarcomas reported a three-year event-free survival (EFS) of 27% and an OS of 34% [6]. Patients with metastasis only to the bone marrow had an EFS of 52%, whereas those with more than five skeletal metastatic lesions had an EFS of just 16% [6]. Another study showed that 43% of patients who received ifosfamide and etoposide combination with methotrexate had three-year event-free survival and did not require supplemental doxorubicin or cisplatin [7].

Chemotherapy is often used to kill the cancer cells. If resectable, surgical removal of the visible tumor can be a definite cure. In many nonresectable Ewing sarcomas, radiation therapy is often used after chemotherapy to further kill cancer cells and control their growth.

Diagnosing ES can be challenging. Utilizing data from clinical, radiological, pathological, and cytogenic sources can provide valuable diagnostic information. In this case report, the patient was diagnosed with ES of the right triceps when he was 12 years old, for which he was treated with chemotherapy and radiation. Patient remained in remission until he was 26 years old. At the age of 26 years, the patient had local recurrence in his right triceps, after which he started on chemotherapeutic regimen and underwent a limb-sparing resection of his right upper arm mass.

Although he was in remission for almost 2.5 years, he had recurrence with metastasis to his lungs, right orbit, and gastrohepatic region. A biopsy specimen was critical in confirming local recurrence of ES.

Overall, we present a unique case of ES that metastasized to the pancreas, which was unresponsive to chemotherapy but had excellent response to radiation therapy. This finding is critical as tumor metastatic to the pancreas could potentially be a poor prognostic indicator. Pancreatic metastases are quite rare, accounting for only 2% of all pancreatic cancers [8, 9]. Most common primary tumors that metastasize to the pancreas are lung cancer, renal cell carcinoma, breast cancer, and melanoma [9].

Cancers of the bone and soft tissue, such as osteosarcoma, chondrosarcoma, Merkel cell carcinoma, and leiomyosarcoma, have also been implicated with metastasis to the pancreas [9, 10]. However, ES metastasis to the pancreas is considered very rare [11].

Based on our literature search in PubMed, 39 cases of ESFT, including ESB, EES, and pPNET involving the pancreas (either as primary or metastasis), were reported.  Table 1 summarizes key diagnostic findings, treatment, and outcome of these previously reported cases. Excluding our case report, only three other cases appear to be metastatic; these patients were treated with a combination of surgical resection, chemotherapy, and radiation. Disease progression and outcome data were unavailable in one of these three cases, as reported by Obuz et al. [12]. The overall prognosis was, however, poor with two of the other reported metastatic cases resulting in death from the disease [10, 13].

Prompt diagnosis, by multimodality approach, which includes appropriate imaging, biopsy (diagnostic or excision) is required to tailor appropriate treatment regimen for optimal benefit. Endoscopic ultrasound (EUS) is both effective and safe for diagnosing pancreatic metastasis [9]. Furthermore, utilizing immunohistochemistry can confirm suspected diagnosis of pancreatic metastasis [9]. CD99 is the most commonly reported marker associated with ES, reported in 31 of the 39 cases found in the literature (Table 2). Other associated markers, but less specific, include neuron specific antigen, vimentin, and synaptophysin.

In our case, IHC stains of his tumor cells were positive for CD99 but negative for CD45, and the tumor cells had similar morphology to those previously found in his right arm sarcoma. Collectively, these diagnostic techniques were critical in supporting the diagnosis of ES metastatic to the pancreas. He was successfully treated with radiation therapy and now remains disease free.

## 4. Conclusion

In conclusion, we present a rare case of ES metastasis to the pancreas in a 29-year-old male with prior diagnosis and treatment of ES of the right triceps. Our case highlights the rarity of ES metastasis to the pancreas, with our literature search only identifying 3 other reported cases in the English literature. Immunohistochemistry stain can greatly aid in diagnosing pancreatic metastasis, with CD99 being the most common marker associated with ES. Our patient responded well to radiation therapy, further highlighting that patients with recurrent ES may have good response to the same therapeutic modality to which they successfully responded in the past. This could be related to the ES tumor genetics and characteristics in the patient and may serve to direct treatment and better improve prognosis.

## Figures and Tables

**Table 1:** Summary of recent cases of Ewing sarcoma FT of the pancreas in the literature.

Author	Age	Gender	Symptom	Pathologic features	Cytogenetics	Primary/metastatic	Location	Size (cm)	Treatment	Progression/outcome	Ref.
Present case	29	M	None	CD99	EWSR1-FLI1 fusion (type 1)	Metastatic—primary at the right tricep with metastases to the right lung, right greater sphenoid, and most recently to the pancreas	Body and neck	1.8 × 1.6	Cyclophosphamide, topotecan, and RAD	12 mos; later; NED	Present case

Nishizawa et al. (2015)	22	M	Upper abdominal pain, nausea, and vomiting	CD99, NSE, VIM, synaptophysin, and neural cell adhesion molecule	22q11 rearrangement	Primary	Pancreatic head	8.5 × 5.0 × 6.2	Whipple, CHE, and RAD	12 mos; AWD	[5]

Rubin et al. (1985)	28	M	None	N/A	N/A	Metastatic—primary ES of the right pubic bone with recurrence in 3 years and metastasis to the pancreas		8 × 6	CHE and RAD	8 mos; died of disease	[10]

Obuz et al. (2000)	15	F	Jaundice	CD99		Metastatic—primary ES in the right hemithorax invading mediastinum	Head	N/A	Partial pancreatic resection	N/A	[12]

Mulligan et al. (1997)	26	M	None	PAS		Metastatic—primary tumor in the left femur and concurrent mass found in the pancreas	Body and tail	N/A	RAD and chemotherapy	15 mos; recurrence, DOD	[13]

Bose et al. (2012)	31	F	Gallstone pancreatitis	CD99 and VIM	EWSR1 gene at 22q12 (FISH)	Primary ES in the pancreas	Posterior junction of the body and tail	3	Distal pancreatectomy, splenectomy, and VAC with alternating IE	18 mos; NED	[14]

Kent et al. (2018)	72	F	Jaundice	CD99 and PAS granules	N/A	Primary	Papilla of Vater	3 × 2 × 1.5	Whipple	2 mos; DOD	[15]

Komforti et al. (2018)	39	M	Abdominal pain	CD99, chromogranin, and synaptophysin	EWSR1-FLI1	Primary	Pancreatic body	8 × 5.8	Pain control	1 mo; AD	[16]

Liu et al. (2018)	36	M	Upper abdominal pain and jaundice	CD99, Ki-67, VIM, *β*-catenin, alpha-1-antichymotrypsin, and S-100	N/A	Primary	Head	6.3 × 3.6 × 4.8	Roux-en-Y choledochojejunostomy, refused RAD and CHE	1 mo; liver metastasis, 2 mos; DOD	[17]

Golhar et al. (2017)	17	F	Jaundice and itching	CD99 and cytokeratin AE1/AE3		Primary	Pancreatic head and uncinate process	5.6 × 7.4	Pancreotoduodectomy, vincristine, cyclophosphamide, and doxorubicin	2 wks; AWD	[18]

Saif et al. (2017)	38	F	Abdominal pain	CD99	t(11;22), (q24; q12)	Primary	Body and tail of the pancreas	8 × 10	Distal pancreatectomy, IE, and VAC	6 mos; AWD	[19]

Teixeira et al. (2015)	28	F	Epigastric pain, jaundice, and pruritus	CD99, VIM, automated CKM, and CD56		Primary	Head and body	13 × 9 × 13	Gastroduodenopancreatectomy	NED postop	[20]

Changal et al. (2014)	60	M	Epigastric pain	CD99, NSE, FLI-1, synaptophysin, and VIM	t(11;22) (q24; q12) via FISH	Primary	Head	3.1 × 2.2	VIDE × 3	3 mos; AWD	[21]

Dias et al. (2013)	25	F	Abdominal pain	CD99 and cytokeratin 8	t(11;22) (q24; q12) translocation	Primary	Head	4.2 × 4.0	Pancreaticoduodenectomy, VAI, and VDC	8 mos; DOD	[22]

Jayant et al. (2013)	20	F	LUQ pain	CD99		Primary	Body and tail	8 × 10	Distal pancreatectomy and splenectomy, VAC, and RAD	24 mos; DOD	[23]

Mao et al. (2013)	13	F	Abdominal pain, anorexia, polyuria, and polydipsia	CD99 and NSE	EWSR1-FLI1 fusion	Primary	Head	9 × 11 × 17	Surgical resection of uncinate process, RAD, VAC, and MAID	9 mos; metastasis to the liver, 36 mos; metastasis to the right kidney; 41 mos; metastasis to the 3^rd^ vertebra, DOD	[24]

Rao et al. (2012)	47	F	Abdominal pain	CD99		Primary	Body and tail	12 × 15	Distal pancreatectomy and splenectomy, IE, and VAC	NED after 2 cycles	[25]

Reilly et al. (2013)	23	M	Upper abdominal pain, nausea	CD99, cytokeratin AE1/AE3, Cam 5.2, CK19, VIM, BCL-2, NSE, EMA, cyclin D1, and PAS granules	EWSR1-FLT1	Primary	Distal body and proximal tail	5.8 × 5.4	Distal pancreatectomy and splenectomy	Unknown	[26]

Jing et al. (2011)	24	F	None	N/A	N/A	Primary	Head	10 × 10 × 8	Resection and CHE	N/A	[27]

Maxwell et al. (2011)	11	M	Fatigue	CD99, broad-spectrum cytokeratin, and VIM	EWSR1-ERG fusion (RT-PCR)	Primary	Head	9.8 × 7.8 × 6.4	VDC alternating with IE, Whipple	3 mos; AWD	[28]

Wakao et al. (2011)	3	M	Upper abdominal pain and swelling	N/A	N/A	N/A	Head	N/A	Pancreatoduodenectomy, VDC/IE, AST, and RAD	8 mos; AWD	[29]

Doi et al. (2009)	37	M	Jaundice	CD99, VIM, CD56, and NSE	EWSR1 rearrangement at 22q12	Primary	Head	6	Pancreatoduodecotomy, hepatic resection, VDC × 7 alternating with IE, RAD	12 mos; NED	[30]

Menon et al. (2006)	8	F	Abdominal pain, menstrual bleeding, breast development, and pubic hair	CD99	N/A	Primary	Body	10 × 6 × 10	Cholecystectomy, doxorubicin, and RAD	CR postop, 19; death 2/2 to heart failure	[31]

Schutte et al. (2006)	2	F	Vaginal bleeding, breast development, and pubic hair	Synaptophysin, CD99, chromogranin A, S-100, VIM, estrogen receptor, progesterone receptor, inhibin, and epithelial membrane antigen	N/A	Primary	Body	6 × 4	Distal pancreatectomy, vincristine, adriamycin, and cyclophosphamide (VDC) alternating with cisplatin and etoposide	12 mos; NED	[32]

Welsch et al. (2006)	33	M	Abdominal pain and vomiting	CD99, VIM, NSE, cytokeratin, EMA, synaptophysin, CD56, and CD117	Translocation involving EWS gene at 22q12	Primary	Body and tail	18 × 18 × 16	Partial gastric resection, left pancreatic resection, splenectomy, VIDE × 6, VAI × 1, and AST	1 mo; metastases to the liver, 12 mos, NED	[33]

Perek et al. (2003)	31	M	RUQ pain	CD99, VIM, Leu 7, and synaptophysin		Primary	Head and body	10 × 12	Whipple, AI × 6, ifosphamide × 6, doxorubicin, and docetaxel	4 mos; local recurrence, 24/36 mos; metastasis to the right lung, 50 mos; DOD	[34]

Takeuchi et al. (2003)	10	F	Upper abdominal pain	N/A	N/A	N/A	Body	N/A	CHE and AST	3 mos; DOD	[35]

Gemechu et al. (2002)	17	M	Abdominal swelling	Synaptophysin and chromogranin		Primary	Body	N/A	N/A	36 mos; AWD	[36]

Movahedi-Lankarani et al. (2002)	17	M	Abdominal pain and jaundice	CD99, cytokeratin AE1/AE3, and NSE	t(11;22) (q24; q12)	Primary	Head	9.0	Whipple and CHE	33 mos; NED	[37]
	20	M	Abdominal pain and jaundice	CD99, cytokeratin AE1/AE3, NSE, and epithelial membrane antigen	t(11;22) (q24; q12)	Primary	Head	3.5	Whipple	27 mos; AWD	[37]
	25	F	Abdominal pain	CD99 and cytokeratin AE1/AE3	N/A	Primary	Head	N/A		N/A	[37]
	21	F	Abdominal pain	CD99, cytokeratin AE1/AE3, NSE, chromogranin, synaptophysin, and epithelial membrane antigen	t(11;22) (q24; q12)	Primary	Head	N/A	Whipple	Death due to OR complication	[37]
	25	F	Abdominal pain and jaundice	CD99 and NSE		Primary	Head	8.0		N/A	[37]
	13	M	Abdominal pain	CD99 and NSE	N/A	Primary	Head	6.0	VDC	43 mos; NED	[37]
	6	M	Abdominal pain and jaundice	CD99, cytokeratin AE1/AE3, NSE, and synaptophysin	t(11;22) (q24; q12)	Primary	Head	3.5	Whipple and VDC	48 mos; recurrence, DOD	[37]

O'Sullivan et al. (2001)	20	M	None	CD99, VIM cytokeratin, and membrane antigen	EWS-7 to Fli1-5	Primary	Head	3.5	Pancreatoduodenectomy, CHE, and RAD	Metastasis to the lungs at 30 mos, AWD	[38]

Bulchmann et al. (2000)	6	F	Paleness, dizziness, and fatigue	CD99, pancytokeratin, NSE, gamma-enolase and squamoid corpuscles, and S-100	Loss of cosmids F7 and E4 distal EWSR1 breakpoint	Primary	Head	4.0 × 5.4 × 3.0	Whipple and colon segmentectomy (refused CHE)	6 mos; recurrence, DOD	[39]

Luttges et al. (1997)	13	F	Dyspepsia and exophthalmos	CD99 and NSE		N/A	Body and tail	22 × 8 × 10	Whipple and CHE	2 mos; AWD	[40]
	31	M	Upper abdominal pain	CD99, NSE, and vimentin		N/A	Body	N/A	CHE and resection	N/A	[40]

Danner et al. (1994)	17	M	Upper abdominal pain	Cytokeratin, NSE, and 12E7	t(11;12) (q24; q12)	Primary	Head	N/A	Pancreatoduodenectomy, VDC/cisplatin+etoposide, and RAD	33 mos; AWD	[41]

AI = adriamycin (doxorubicin)/ifosfamide; IE = ifosphamide and etoposide; VAC = vincristine, adriamycin, and cyclophosphamide; VDC = vincristine, doxorubicin and cyclophosphamide; RAD = radiation; VIDE = vincristine, ifosfamide, doxorubicin, and etoposide; VAI = vincristine, actinomycin D and ifosfamide; CR = complete remission; AST = autologous stem transplant; MAID = doxorubicin/dacarvazine/ifosfamide; AWD = alive without disease; DOD = died of disease; DOC = died of complications; AD = alive with disease; NED = no evidence of disease; CHE = chemotherapy (specific agents unknown); NSE = neuronspecific antigen; VIM = vimentin.

**Table 2:** Summary of markers in 39 ESFT cases with pancreatic involvement reported to date.

Marker	Number of times reported positive
CD99	31 (78%)
Neuron specific enolase (NSE)	16 (40%)
Vimentin	12 (30%)
Synaptophysin	9 (23%)
Cytokeratin AE1/AE3	7 (18%)
Chromagranin	4 (10%)
S-100	4 (10%)
Epithelial membrane antigen	4 (10%)
PAS	3 (7.5%)
CD56	3 (7.5%)
Neural cell adhesion molecule	1 (2.5%)
FLI-1	1 (2.5%)
Cytokeratin 8	1 (2.5%)
CD45	1 (2.5%)
Alpha 1 antichromotrypsin	1 (2.5%)
CKM	1 (2.5%)
Progesterone receptor	1 (2.5%)
Estrogen receptor	1 (2.5%)
Inhibin	1 (2.5%)
Cyclin D	1 (2.5%)
Leu 7	1 (2.5%)
BCL-2	1 (2.5%)
CK 19	1 (2.5%)
CAM 5.2	1 (2.5%)
*β*-Catenin	1 (2.5%)
12E7	1 (2.5%)
